# Aging-enhanced autophagy activity promotes fibrotic progression via the TGF-β2/Smad signaling pathway in trabecular meshwork cells—a new insight from POAG

**DOI:** 10.3389/fmed.2024.1534120

**Published:** 2025-01-15

**Authors:** Jin Han, Jun Wang, Ling Shen, Yiting Cai, Xuze Wang, Ailixiati Wumaier, Wei Chen, Wei Han

**Affiliations:** ^1^Eye Center of the Second Affiliated Hospital, Zhejiang University School of Medicine, Hangzhou, Zhejiang, China; ^2^Stomatology Hospital, School of Stomatology, Zhejiang University School of Medicine, Hangzhou, Zhejiang, China; ^3^Institute of Immunology, Zhejiang University School of Medicine, Hangzhou, Zhejiang, China

**Keywords:** POAG, autophagy, aging, trabecular meshwork, fibrosis

## Abstract

**Introduction:**

Glaucoma, a leading cause of irreversible blindness, is characterized by optic neuropathy and retinopathy, with primary open-angle glaucoma (POAG) being the most prevalent form. The primary pathogenic mechanism of POAG involves elevated intraocular pressure caused by chronic fibrosis of the trabecular meshwork (TM). Autophagy, a critical process for maintaining cellular homeostasis, has been implicated in fibrosis across various organs. However, its precise role in the fibrosis associated with POAG pathogenesis remains unclear. This study investigates the involvement of autophagy in TM fibrosis and explores its potential impact on POAG development, aiming to provide insights into new therapeutic targets.

**Methods:**

To assess autophagy activity and its relationship with fibrosis, we analyzed TM tissues from POAG patients and healthy donors. Autophagic activity in human TM tissues was measured through immunohistochemical analyses. An in vitro aging model using chronic H_2_O_2_ treatment was established to investigate the change of fibrosis in TM cells. Additionally, we used dexamethasone-treated TM cells as a POAG model to explore the role of autophagy in fibrotic progression. The involvement of the TGF-β2/Smad signaling pathway was investigated through western blot analysis and quantitative real-time PCR.

**Results:**

This study reveals increased autophagic activity in tissues from POAG patients and an age-related upregulation of autophagy in healthy human TM tissues. In the H_2_O_2_-induced aging model, TM cells displayed both elevated autophagic activity and fibrosis. Further investigation showed that enhanced autophagy activity promoted fibrotic progression via activation of the TGF-β2/Smad signaling pathway. Similarly, in the dexamethasone-treated TM cell model, autophagy was found to exacerbate fibrosis, aligning with observations in the aging model.

**Discussion:**

In this study, we uncover the interplay between autophagy and the TGF-β2/Smad pathway in the pathogenesis of POAG. We observed increased autophagic activity in TM tissues from POAG patients and in TM tissues of aging healthy individuals. In human primary TM cells, we confirmed that autophagy becomes activated in the context of cellular senescence and the development of POAG, which further facilitates fibrotic progression via the TGF-β2/Smad signaling pathway. These findings underscore the important role of autophagy in POAG pathogenesis and confirm senescence as a pivotal risk factor.

## 1 Introduction

Glaucoma, a leading cause of irreversible vision loss, constitutes a group of neurodegenerative diseases that affect over 70 million individuals worldwide (1). One of the most prevalent forms of glaucoma is primary open-angle glaucoma (POAG), characterized by an open iridocorneal angle with elevated intraocular pressure (IOP) and optic nerve damage (2, 3). The primary pathogenic mechanism of POAG is the elevation of intraocular pressure due to the chronic fibrosis of the trabecular meshwork (TM), a complex three-dimensional structure composed of trabecular meshwork cells (TMC) and a multi-layered extracellular matrix (ECM) secreted by TMC (4, 5).

Accumulated fibrotic proteins in the TM of POAG patients, including fibronectin (FN), collagen, and alpha-smooth muscle actin (αSMA), are known to form a special structure named sheath-derived (SD)-plaques. SD-plaques have been revealed to be markedly more prevalent in glaucomatous eyes than in healthy ones through ultrastructural assessment. The presence of SD-plaques within the TM leads to an increase in its stiffness, thereby contributing substantially to the elevated IOP in POAG (6). In POAG, levels of transforming growth factor-β2 (TGF-β2) are significantly elevated in the aqueous humor (AH), correlating with disease progression (7, 8). TGF-β2 is a central cytokine that significantly advances the fibrotic process by inducing a secretory and myofibroblast-like phenotype in cultured human TM cells. Ocular tissues, such as the TM, iris, and ciliary body, locally secrete TGF-β2, which engages with a rich array of its receptors predominantly situated in the TM. TGF-β2 signaling to the nucleus predominantly involves the phosphorylation of cytoplasmic Smad family proteins. The receptor-associated Smads, comprising R-Smads and Smad1, 2, 3, 5, and 8, with Smad3 being a key mediator of fibrotic processes, play integral roles in signaling pathways. Phosphorylation of Smad3 leads to increased expression of downstream fibrotic proteins (9, 10).

Autophagy, a highly conserved cellular process for the degradation and recycling of damaged components, exhibits a dual influence on fibrosis, with the observed effects differing across various fibrotic disorders. In the liver, increased autophagic activity in hepatic stellate cells was found to be associated with the activation of hepatic stellate cells and hepatic fibrogenesis. A similar pattern of autophagy activation is implicated in the pathogenesis of pulmonary fibrosis at various levels (10–12). In contrast, autophagy has been demonstrated to exert a protective effect against renal epithelial cell fibrosis, highlighting the complex and context-dependent roles of autophagy in fibrotic diseases. However, TM fibrosis, a determinant in the pathological processes of POAG, has an undefined relationship with autophagy. Previous research on autophagy in POAG has largely focused on the optic nerve and retinal ganglion cells (RGCs). A recent study conducted on the spontaneous ocular hypertensive DBA/2 J mice suggested that overactivation of autophagy by expression of the GFP-LC3 transgene leads to optic nerve degeneration. Another study has shed light on the protective role of autophagy in RGCs by administering autophagy activators through a single intravitreal injection in a rat model of glaucoma induced by polystyrene microbeads (11, 12). Studies exploring the TM-autophagy link have predominantly been conducted in POAG-free conditions. The initial research focused on the assessment of autophagy in TM specimens from human biopsies and found that autophagy levels are significantly heightened in TM, particularly in subjects aged over 60 years (13). Of note, the aforementioned study did not incorporate any samples from patients with POAG (13). Other investigations into the influence of autophagy on the TM were performed *in vitro*, emphasizing the significance of autophagy in modulating TM cell function and maintaining cellular homeostasis against mechanical stress. When TM cells are exposed to mechanical stretch, the mechanical stress-induced autophagy may contribute to modify and reinforce the TM cell cytoskeleton (14). *In vitro* studies examining the effect of autophagy on the viability of TM cells have yielded conflicting results, with autophagy activation reported to either reduce or enhance cell viability (15, 16). Therefore, the involvement of autophagy in TM fibrosis merits further investigation.

Population-based studies have established senescence as a significant risk factor for developing POAG (17–20). Morphological and biochemical analyses of the TM of POAG patients revealed cellular senescence, cell losses, increased accumulation of extracellular matrix (ECM), and the process of subclinical inflammation (21, 22). Moreover, the amount of SD-plaques we mentioned before also increased with age (23). Cellular senescence can be classified into several types, including stress-induced premature senescence (SIPS), replicative senescence (RS), and oncogene-induced senescence (24). It has been suggested that the accumulation of aging cells in POAG with age may mainly related to SIPS, rather than RS (21). Senescent cells secrete numerous proteins collectively designated the senescence-associated secretory phenotype (SASP), encompassing diverse growth factors, proteases, chemokines, and cytokines (25–29). These aging-related changes are often accompanied by fibrosis, and the increased stiffness related to TM fibrosis occurs concomitantly with aging (30, 31). Among the molecular changes associated with aging, altered autophagy has been recognized as a common feature across various organs. While the decline of autophagy underlies aging and disease phenotypes, excessive autophagy may also contribute to the deterioration of cellular function in some contexts (32). Therefore, we aim to elucidate the interplay among aging, autophagy, and TM fibrosis in the pathogenesis of POAG.

In this study, autophagy was identified as playing a key role in POAG pathogenesis. We observed increased autophagic activity in TM tissues from POAG patients and noted an age-related upregulation of autophagy in healthy human TM tissue. Chronic H_2_O_2_ treatment, serving as an aging model, induced increases in autophagy and fibrosis in TM cells. Further analysis revealed that enhanced autophagy activity accelerates fibrotic progression through the TGF-β2/Smad signaling pathway. By utilizing dexamethasone (DEX)-treated TM cells to mimic POAG, we confirmed the pro-fibrotic role of autophagy. Our findings could offer valuable knowledge for enhancing comprehension of fibrogenesis in POAG, potentially unveiling novel avenues for therapeutic intervention.

## 2 Materials and methods

### 2.1 Tissue sampling

Our study group consisted of 16 eyes belonging to 4 POAG patients (mean age ± SD，79.1 ± 5.5 years; male/female, 2/2) and 12 healthy corneal donors (mean age ± SD, 61.75 ± 27.1 years; male/female, 7/5) from the Second Affiliated Hospital of Zhejiang University. Patients with secondary etiologies, such as pseudoexfoliation or pigmentary glaucoma, were excluded from the study. TM samples were taken from human scleral rings collected immediately after donor death. The study was approved by the Research Ethics Committee of The Second Affiliated Hospital, Zhejiang University School of Medicine. The study’s ethical issues also conform to the Declaration of Helsinki.

### 2.2 Immunohistochemistry

Human trabecular meshwork tissues were fixed and embedded in paraffin for immunohistochemistry analysis. Tissues were sliced, de-waxed, and rehydrated. Sections were then blocked with 5% normal goat serum and incubated with primary antibody (LC3B rabbit pAb, ab48394) overnight at 4°C, followed by biotinylated secondary antibody for 2 h at room temperature. Images were acquired using an Olympus BX61 microscope. Three randomly selected images from each sample were analyzed to determine the staining intensity index of LC3 proteins. The cells were categorized and scored according to the intensity of staining: (i) negatively stained, each cell scored 0; (ii) mildly stained, each cell scored 1; (iii) moderately stained, each cell scored 2; and (iv) strongly stained, each cell scored 3. The staining intensity index was calculated as the aggregate score divided by the cell number.

### 2.3 Cell culture and treatment

Primary human trabecular meshwork cells (HTMCs) were purchased from ScienCell Research Laboratories (Carlsbad, United States). Cells were cultured in TM Cell Medium consisting of 500 mL of basal medium, 10 mL of fetal bovine serum (FBS; Gibco, Gaithersburg, MD), 5 mL of TM cell growth supplement (Cat. No. 6592, ScienCell), and 5 mL of penicillin/streptomycin solution. Cells were cultured at 37°C in an atmosphere containing 5% CO_2_. Cells were inoculated in 6-well cell culture plates at a density of 1 × 10^5^ cells per well at 12 h before treatment. Cells were treated with 100 μM H_2_O_2_ or 100 nM dexamethasone (D-085 Sigma) once a day, with or without co-treatment of 2 μM rapamycin (S1039, Selleck), 2 mM 3-methyladenine (GC10710, GLPBIO) for 24 h.

### 2.4 Western blot analysis

Cultured cells were collected and lysed with iced NP-40 lysis buffer (P0013F, Beyotime) containing phosphatase inhibitors (4,906,837,001, Roche) and phenylmethylsulfonyl fluoride (PMSF) (ST506, Beyotime). The protein samples were separated by sodium dodecyl sulfate-polyacrylamide gel-electrophoresis and transferred to polyvinylidene fluoride membranes (Immobilon-P, IPVH00010). The membranes were blocked for 2 h at room temperature in Tris-buffered saline containing 5% skim milk and 0.05% Tween-20 and then incubated with primary antibodies overnight at 4°C. The membranes were washed and incubated with secondary antibodies for 2 h at room temperature. Subsequently, the membranes were scanned using an Alpha Chemiluminescence Gel Imaging System (FluorChem E, Cell Biosciences). All experiments were repeated thrice, and the images were analyzed using ImageJ software.

The primary and secondary antibodies are listed in Table 1.

**Table 1:** Antibodies used for Western blot.

Antibodies	Company	Catalog no.	Dilution
LC3B rabbit mAb	Abcam	ab192890	1:2,000
SQSTM1/p62 mouse mAb	Abcam	ab56416	1:2,000
αSMA rabbit pAb	Abcam	ab5694	1:1,000
Fibronectin rabbit mAb	Abcam	ab45688	1:2,000
Smad3 rabbit mAb	Abmart	T55013	1:1,500
Phospho-Smad3 Antibody	Abmart	T55140	1:1,500

### 2.5 Quantitative real-time PCR

Total RNA was extracted from TM cells using TRIzol reagent (Invitrogen, 10,296,010), and quantified with NanoDrop2000 (Thermo). Reverse transcription was performed with a PrimeScriptTM RT reagent Kit with gDNA eraser (Cat.# RR047A, Takara, Japan) according to the manufacturer’s instructions. After 24 h of culture, the culture medium of TM cells plated in 6-well plates was replaced with fresh medium. Next, cells were treated with 100 μM H_2_O_2_ for 4 days. Cells cultured with fresh complete medium were used as control. Cells were collected and centrifugally precipitated, and total RNA was extracted by Trizol (Invitrogen). The purity and concentration of RNA were determined based on OD260/280 ratios. Reverse transcription was performed by using a HiScript III All-in-one RT SuperMix (Vazyme; R333), following the manufacturer’s instructions. Relative mRNA levels of genes of interest were determined by quantitative real-time PCR (qRT-PCR). GAPDH was used as an internal control. All experiments were performed in triplicate and repeated at least three times (Table 2).

**Table 2:** Gene expression analysis by qPCR.

Gene	Forward 5′–3′	Reverse 5′–3′
FN	ACGACTGTGGACCAAGTTGATGAC	TGAGTTCTGTGCTGCTACCTTCTAC
αSMA	GGTCGGTATGGGTCAGAAAGATTC	GCTCGTTGTAGAAGGTGTGGTG
IL-8	ACCACACTGCGCCAACACAG	AACTTCTCCACAACCCTCTGCAC
IL-1β	ATGGCTTATTACAGTGGCAATGAGG	AGTGGTGGTCGGAGATTCGTAG
IL-6	TTCGGTCCAGTTGCCTTCTCC	TCTGAAGAGGTGAGTGGCTGTC
TNF-α	AATGGCGTGGAGCTGAGAGATAAC	CGGCTGATGGTGTGGGTGAG
TGF-β2	GAGTGCCTGAACAACGGATTGAG	GCCATTCGCCTTCTGCTCTTG
CXCL11	TGCTACAGTTGTTCAAGGCTTCC	GCTTTCTCAATATCTGCCACTTTCAC
PAI-1	CCACCGCCGCCTCTTCC	GCAGTTCCAGGATGTCGTAGTAATG
MMP3	TGATGAACAATGGACAAAGGATACAAC	AGGTCTGTGAGTGAGTGATAGAGTG
MMP2	ACCTACACCAAGAACTTCCGTCTG	TGCCAAGGTCAATGTCAGGAGAG

### 2.6 CCK8 assay

The CCK8 assay was performed to assess cell viability. TM cells were inoculated in 96-well cell culture plates at a density of 2 × 10^3^ cells per well for 12 h before exposure to the stimulus. Each well was added with 10 μL CCK8 solution (GK10001, GLPBIO) and incubated at 37°C for 2 h. The absorbance was measured by a Thermo Scientific Varioskan Flash at 450 nm. All experiments were performed in triplicate and repeated at least three times.

### 2.7 Senescence-associated *β*-galactosidase assay

The assay was performed using the SA-β-gal Staining Kit (Cat.# C0602, Beyotime, Shanghai, China). In brief, the cells were washed, fixed, and incubated overnight at 37°C with a staining solution containing X-gal. Then, the staining was imaged and quantified under a regular light microscope.

### 2.8 Immunofluorescence microscopy

TM cells were seeded on coverslips placed in 24-well plates. After treated with stimulus for the indicated time, cells were fixed in 4% paraformaldehyde for 15 min at room temperature and washed three times with PBS. The fixed cells were then permeabilized with 0.1% TritonX-100 (Cat# 85111, Thermo Scientific) in PBS for 20 min and blocked with 10% goat serum in PBS for 2 h. The cells were incubated with primary antibody (LC3 rabbit, Abcam; SQSTM1/p62 mouse, Abcam;) for 12 h at 4°C, and then incubated with secondary antibody [goat anti-mouse IgG (H&L) (Alexa Fluor 546), A-11003, Invitrogen; goat anti-rabbit IgG (H&L) (Alexa Fluor 488), A-11008 Invitrogen] for 2 h at room temperature. The cells were rinsed again and stained with DAPI (D1306, ThermoFisher). Samples were observed using a confocal microscope system (IX83-FV3000-OSR, Olympus).

### 2.9 Statistical analysis

Statistical analysis was performed by SPSS software (version 26.0, IBM Corp.). Data were presented as mean ± standard error of the mean (SEM). For comparisons between the two groups, Student’s *t*-test was performed. A one-way analysis of variance (ANOVA) followed by the Bonferroni *post hoc* test was performed for comparisons among more than two groups. All statistical tests were two-sided. *p* < 0.05 was considered statistically significant. All experiments were carried out at least three times unless otherwise stated.

## 3 Results

### 3.1 Trabecular meshwork tissue from POAG patients displays augmented autophagic activity

**Figure 1:**
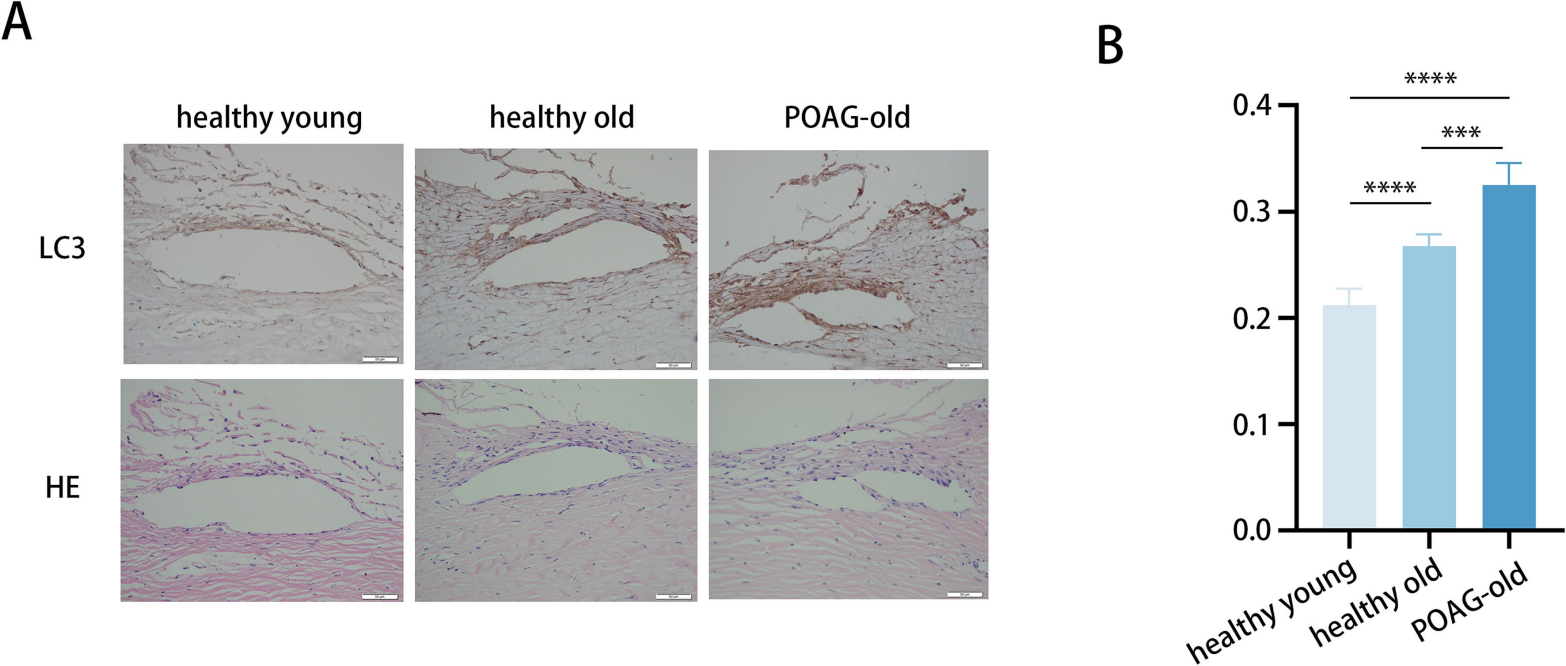
Trabecular meshwork tissue from POAG patients displays augmented autophagic activity. **(A)** Representative immunohistochemistry images of TM tissues from 6 healthy young individuals (41.3 ± 6.7), 6 healthy elders (82.3 ± 3.4), and 4 POAG patients (79.0 ± 2.2). Scale bar, 50 μm. **(B)** Staining intensity index of LC3 proteins from 16 corneal donors. Mean ± SD; ***p < 0.001, ****p < 0.0001; (one-way ANOVA).

We collected TM specimens from 16 corneal donors; 4 suffered from POAG, whereas 12 were healthy and devoid of ocular pathologies. These specimens were categorized into three groups: healthy donors aged 30–50 (41.3 ± 6.7), healthy donors aged 70–90 (82.3 ± 3.4), and POAG patients aged 70–90 (79.0 ± 2.2) (Figure 1A).

To assess the potential role of autophagy in POAG pathogenesis, the expression levels of LC3 in these TM specimens were quantified (Figure 1B). The immunohistochemistry staining demonstrated markedly elevated LC3 protein levels in TM of POAG patients versus age-matched controls. Moreover, LC3 protein levels in older healthy individuals were higher compared to younger healthy individuals. Additionally, representative images showing the anatomic structures of the anterior segment (Lower panels: H&E staining). Age-related anatomic changes in the trabecular meshwork (TM) include the accumulation of extracellular matrix, thickening of the TM, and a reduction in the number of TM cells. Aging-related alterations frequently coincide with fibrosis, and the augmented stiffness caused by TM fibrosis occurs concomitantly with aging (30, 31). Consequently, to gain a deeper understanding of how autophagy activity and aging process influences fibrosis in the TM, thereby contributing to the development of POAG, we employed an *in vitro* model utilizing primary human TM cells for a comprehensive analysis.

#### 3.1.1 Chronic oxidative stress upregulates fibrosis and autophagy in TM cells

Considering the significant relationship between cellular dysfunction associated with aging and fibrosis, it is reasonable to examine TM fibrosis using a model that recapitulates the aging TM cell phenotype (33, 34). Existing studies demonstrate that an escalation in reactive oxygen species (ROS), attributable to oxidative stress, exerts a substantial impact on accelerating the aging process of TM cells (35). Thus, we adopted a chronic oxidative stress model to mimic *in vitro* aging of TM cells.

**Figure 2:**
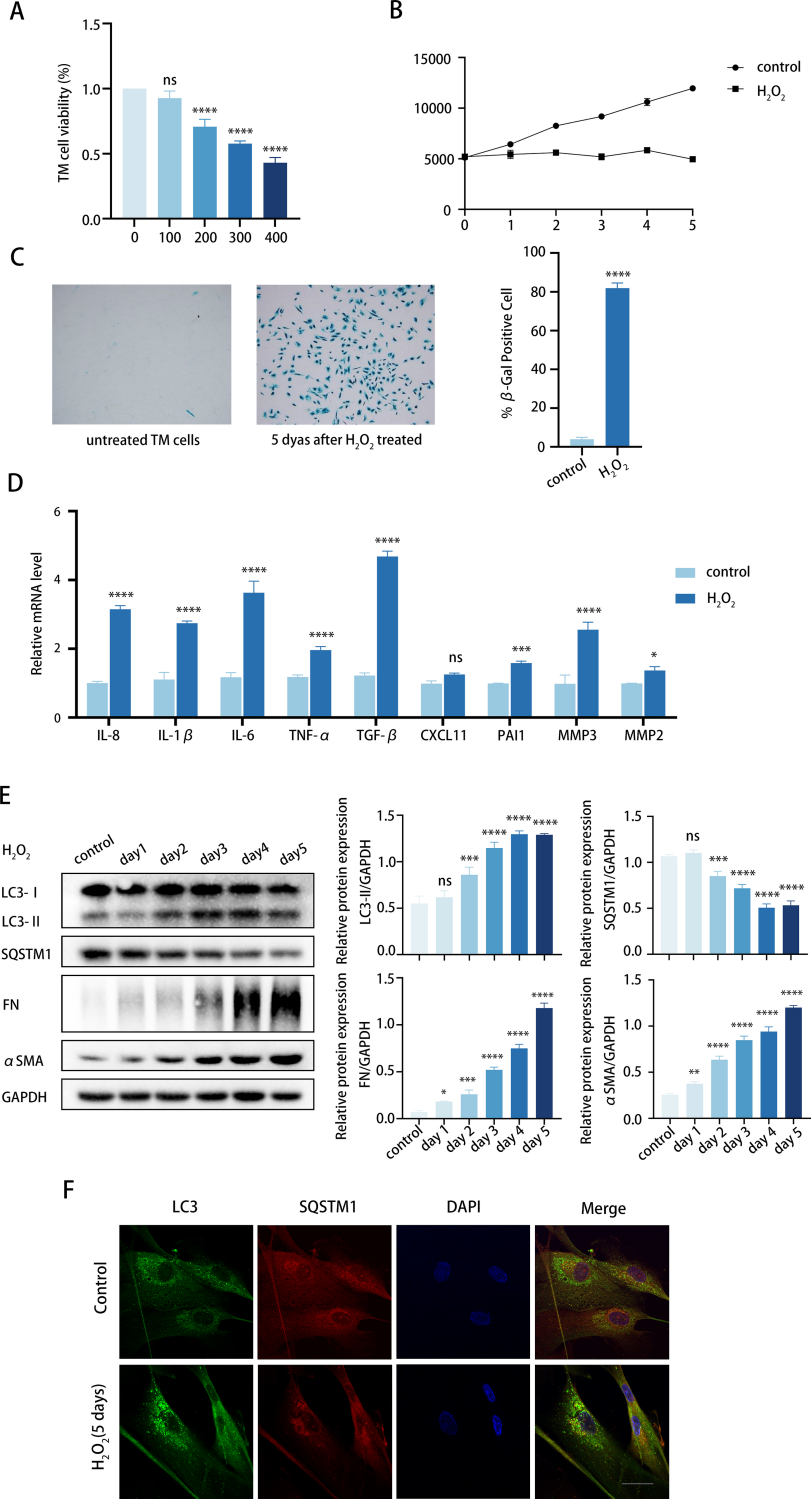
Chronic oxidative stress upregulates fibrosis and autophagy in TM cells. **(A)** Viability of TM cells after being treated with H_2_O_2_ (100–400 μM) daily for 6 days consecutively, determined by the CCK8 assay. **(B)** Growth curve of both H_2_O_2_-treated and untreated cells. Cell number was determined daily after cell exposure to 100 μM H_2_O_2_. **(C)** Senescent cells identified by SA-β-Gal. Quantitative assay of SA-𝛽-Gal-positive cells (right). Data are presented as Mean ± SD from three independent experiments (****p < 0.0001 vs. control group; unpaired Student’s *t*-test). **(D)** The SASPs including IL-8, IL-1β, IL-6, TNF-*α*, TGF-β2, CXCL11, PAI1, MMP3, and MMP2 were detected using RT-PCR (ns, not significant; *P < 0.05, ***p < 0.001, ****p < 0.0001 vs. control group; paired Student’s *t*-test). **(E)** The protein levels of LC3, SQSTM1, FN, and αSMA were detected by a Western blot. GAPDH was used as an internal control. Quantified protein levels were displayed as bar graphs (right). Data are presented as Mean ± SEM from three independent experiments (ns, not significant; *P < 0.05, **P < 0.01, ***p < 0.001, ****p < 0.0001 vs. control group; paired Student’s *t*-test). **(F)** Confocal images of LC3 (green), SQSTM1 (red), and DAPI (blue). Scale bar, 50 μm.

Initially, we investigated the impact of culture conditions on inducing senescence in TM cells without causing cell death. TM cells were daily treated with H_2_O_2_ at concentrations ranging from 100 to 400 μM for 6 consecutive days. Cell treatment with 100 μM H_2_O_2_ showed no cytotoxicity, whereas concentrations of 200, 300, and 400 μM were cytotoxic, according to the CCK8 assay (Figure 2A). Next, we investigated whether cumulative 100 μM H_2_O_2_ treatment induced premature senescence of TM cells. To achieve this, we examined a variety of senescent-associated biomarkers (36), including senescence-associated *β*-galactosidase (SA-β-Gal) staining, cell proliferation, and SASP secretion.

To determine whether the treatment induced permanent growth arrest, we analyzed the proliferative capacity of the cells according to the CCK-8 assay (37). As illustrated in Figure 2B, the growth curve exhibited a twofold increase in proliferating control cells within 5 days, whereas the curve was significantly suppressed in the H_2_O_2_-treated cells. Given that 100 μM H_2_O_2_ did not significantly reduce TM cell viability (Figure 2A), we postulated that H_2_O_2_-induced growth inhibition might be due to cell cycle arrest rather than cell death. Meanwhile, senescent cells showed increased SA-*β*-Gal staining over time, with peak positivity observed at 5 days post-treatment, affecting over 80% of cells (Figure 2C). Furthermore, H_2_O_2_ upregulated the expression of multiple SASPs in TM cells, such as TGF-β2, IL-6, IL-8, TNF-*α*, IL-1β, CXCL11, PAI1, and MMP3 (Figure 2D). Notably, TGF-β2 and IL-6, known for their roles in fibrosis, exhibited a significant increase relative to other cytokines. It can be concluded that chronic H_2_O_2_ stress induced premature senescence in TM cells.

Given the concurrent rise in TM stiffness due to fibrosis and advancing age, and our observation of elevated autophagy activity in TM specimens from elderly individuals, we proceeded to investigate the markers of fibrosis and autophagy within our aging model. We analyzed the expression of fibrotic proteins. FN expression, an essential component of ECM, rose with prolonged H_2_O_2_ exposure. Similarly, αSMA protein levels, indicative of TM stiffness, also increased (Figure 2E). This suggests that the fibrosis level of TM cells was upregulated while aging. Meanwhile, the LC3-II/GAPDH ratio increased progressively, while SQSTM1 protein levels decreased with H_2_O_2_ treatment. Additionally, the immunofluorescence assay demonstrated that the number of LC3 puncta markedly increased after 5 days of H_2_O_2_ exposure compared to the control group (Figure 2F). It is noteworthy that the alterations in FN and αSMA exhibited a progressive pattern that correlated with increased autophagy levels. Thus, we would like to further determine whether autophagy is involved in the regulation of TM fibrosis.

#### 3.1.2 Autophagy promotes fibrotic processes in TM cells

As previously stated, we found FN and αSMA levels increased in tandem with autophagy (Figure 2F). The exact effect of autophagy on TM cell fibrosis was investigated via elevating the autophagic flux by rapamycin (Rapa) and downregulating the autophagic flux by 3-methyladenine (3-MA).

**Figure 3:**
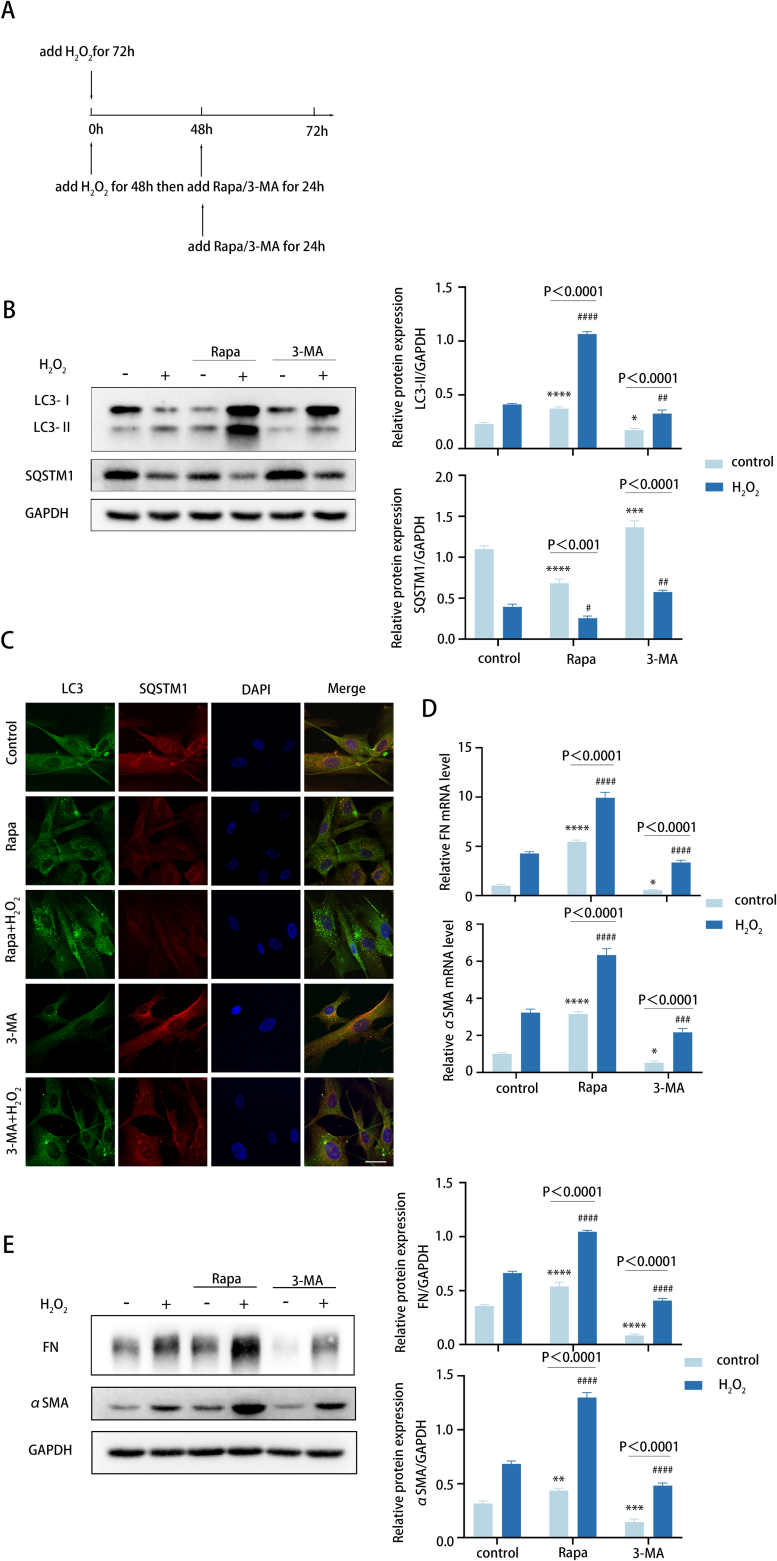
Autophagy promotes fibrotic processes in TM cells. **(A)** Different treatments are described as follows. (i) Cells were treated with H_2_O_2_ (100 μM) for 72 h. (ii) Cells were treated with H_2_O_2_ (100 μM) for 48 h and then co-treated with Rapa (2 μM) or 3-MA (2 mM) for 24 h. (iii) Cells were only treated with Rapa (2 μM) or 3-MA (2 mM) for 24 h. **(B)** The protein levels of LC3 and SQSTM1 were detected by a Western blot. GAPDH was used as an internal control (left). Quantified protein levels were displayed as bar graphs (right). **(C)** Confocal images of LC3 (green), SQSTM1 (red), and DAPI (blue). Scale bar, 50 μm. **(D)** The mRNA expression levels of FN and αSMA were detected using RT-PCR. **(E)** The protein levels of FN and αSMA were detected by a Western blot. GAPDH was used as an internal control. Quantified protein levels were displayed as bar graphs (right). All quantitative data are presented as Mean ± SEM from three independent experiments (*P < 0.05, **P < 0.01, ***p < 0.001, ****p < 0.0001 vs. control group; #*P* < 0.05, ##*P* < 0.01, ###*p* < 0.001, ####*p* < 0.0001 vs. H_2_O_2_-treated group; one-way ANOVA) Rapa, rapamycin; 3-MA, 3-methyladenine.

To determine the role of autophagy in TM cell fibrosis, we examined the expression of FN and αSMA in TM cells both in normal TM cells and the aging TM cells treated with H_2_O_2_, rapamycin combined with H_2_O_2_, and 3-MA combined with H_2_O_2, respectively,_ (Figure 3A). In TM cells, 72 h H_2_O_2_ treatment moderately elevated autophagic activity. An additional 24-h rapamycin treatment further elevated the autophagic activity of TM cells that were pre-treated with H_2_O_2_ for 48 h, with elevated LC3-II/GAPDH ratio and reduced SQSTM1 protein levels (Figure 3B). Conversely, a 24-h treatment with 3-MA markedly suppressed autophagy level (Figure 3B). Consistently, Rapa combined with H_2_O_2_ treatment enhanced the accumulation of LC3 puncta in TM cells compared with 72 h H_2_O_2_ treatment, whereas 3-MA combined with H_2_O_2_ treatment inhibited LC3 puncta accumulation (Figure 3C).

For TM fibrosis, the effects of rapamycin and 3-MA on the expression level of FN and αSMA were assessed, including the mRNA and protein levels. Strikingly, rapamycin combined with H_2_O_2_ treatment dramatically upregulated FN and αSMA expression levels (Figures 3D,E). In contrast, 3-MA combined with H_2_O_2_ treatment led to an obvious decrease (Figures 3D,E). Taken together, these data indicated that autophagy promotes the fibrotic process of TM cells, and this process is aggravated in the aging model.

#### 3.1.3 Autophagy enhances TGF-β2/Smad-mediated TM cell fibrosis

TGF-β2, a pivotal cytokine in the fibrotic process, was demonstrably elevated in various fibrotic tissues. TGF-β2 binding to its receptors results in phosphorylation of Smad2 and Smad3, which subsequently partner with Smad4 and translocate into the nucleus to form transcriptional complexes that regulate the expression of target genes, including FN and αSMA (38). Smad3 is a key mediator of fibrotic processes (39). Previous findings have confirmed the presence of elevated intraocular TGF-β2 levels in POAG (40).

**Figure 4:**
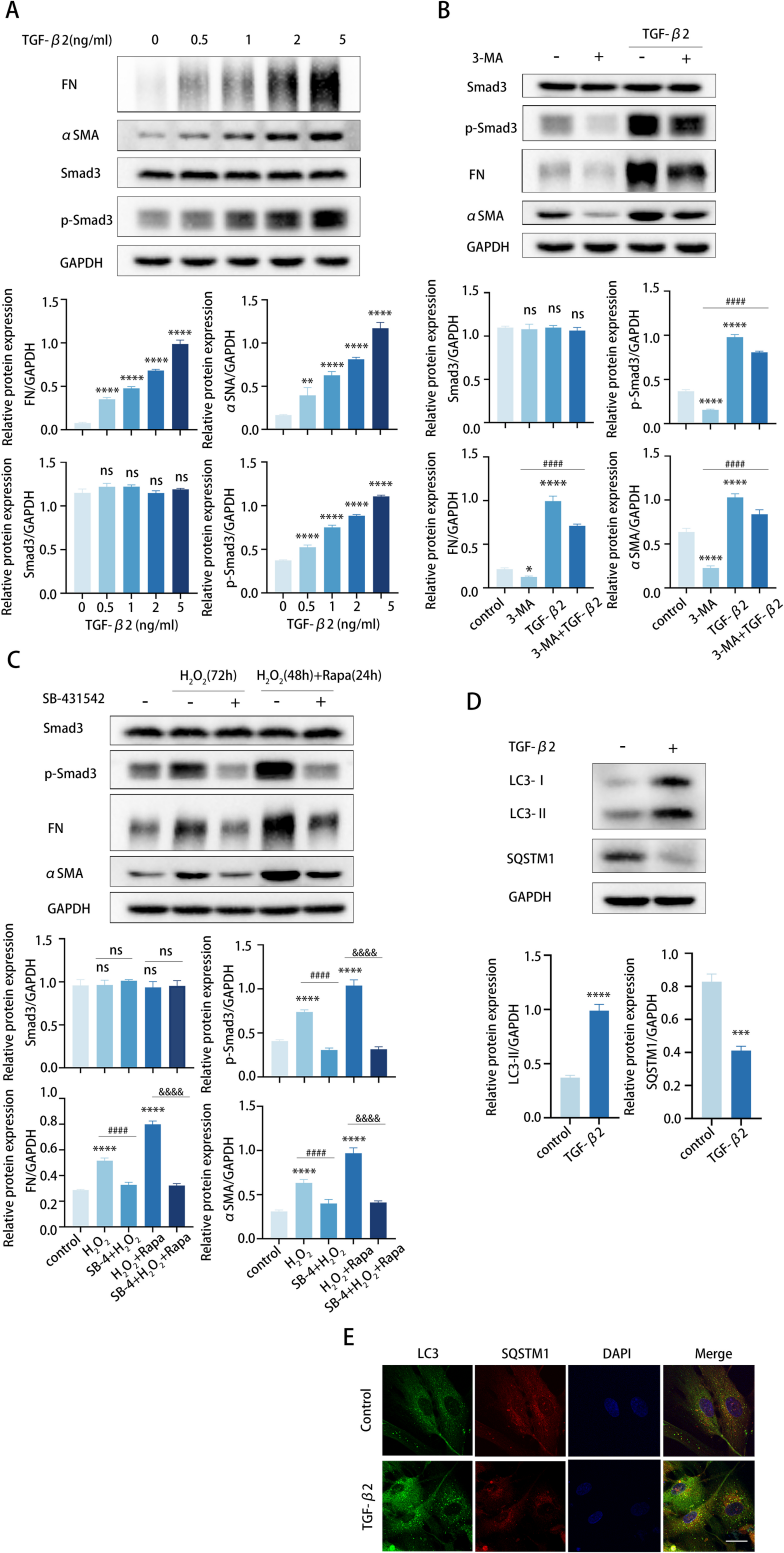
Autophagy enhances TGF-β2/Smad-mediated TM cell fibrosis. **(A–C)** The protein levels of FN, αSMA, Smad3, and p-Smad3 were detected by a Western blot. GAPDH was used as an internal control. Quantified protein levels were displayed as bar graphs (below). **(A)** TM cells were stimulated with different concentrations of TGF-β2 for 48 h. **(B)** TM cells before and after pre-treating with 2 mM 3-MA for 24 h, then incubated in the presence or absence of 5 ng/mL TGF-β2 for 48 h. Data are presented as Mean ± SEM from three independent experiments (*P < 0.05, ***p < 0.001, ****p < 0.0001 vs. control group; ###*p* < 0.001 vs. 3-MA treatment group; one-way ANOVA) Rapa, rapamycin; 3-MA, 3-methyladenine. **(C)** TM cells before and after pre-treating with 10 μM SB-431542 for 2 h, then incubated in the presence or absence of (72 h) H_2_O_2_ or (48 h) H_2_O_2_ plus (24 h) Rapa. Data are presented as Mean ± SEM from three independent experiments (ns, not significant; ****p < 0.0001 vs. control group; ####*p* < 0.0001 vs. H_2_O_2_-treated group; &&&&*p* < 0.0001 vs. H_2_O_2_-Rapa cotreatment group; one-way ANOVA) Rapa, rapamycin; SB-4, SB-431542. **(D)** TM cells were treated with 5 ng/mL TGF-β2 for 48 h. The protein levels of LC3 and SQSTM1 were detected by a Western blot. GAPDH was used as an internal control. Quantified protein levels were displayed as bar graphs (below). **(E)** Confocal images of LC3 (green), SQSTM1 (red), and DAPI (blue). Scale bar, 50 μm.

We exposed TM cells to various concentrations of TGF-β2 and assessed TM cell fibrosis. TGF-β2 treatment induced a dose-dependent accumulation of FN and αSMA in TM cells, indicating an increased fibrotic response (Figure 4A). To explore whether autophagy aggravated fibrosis via the TGF-*β*2/Smad pathway, we treated TM cells with 3-MA and investigated the expression of Smad3, pSmad3, FN, and αSMA. Interestingly, we found that 3-MA-induced reductions in pSmad3, FN, and αSMA were reversed by TGF-β2 treatment (Figure 4B).

To further confirm our presumption, we pretreated TM cells with the specific inhibitor SB-431542 to prevent TGF-β2-mediated Smad3 phosphorylation. TM cells were pretreated with SB-431542 for 6 h and subsequently stimulated with H_2_O_2_ or rapamycin for the detection of Smad3, pSmad3, FN, and αSMA expression levels. As expected, SB-431542 successfully hindered the synthesis of FN and αSMA in TM cells, and this hindrance was not counteracted by subsequent H_2_O_2_ or rapamycin treatment (Figure 4C). In aggregate, these data revealed that the elevated fibrosis induced by autophagy mainly through the TGF-β/Smad signaling pathway. In addition, TGF-β2 may also subtly promote autophagy in TM cells, as suggested by an elevated LC3-II/GAPDH ratio, greater formation of LC3 puncta, and lower levels of SQSTM1 protein (Figures 4D,E).

#### 3.1.4 Autophagy inhibition attenuates fibrosis induced by DEX treatment

**Figure 5:**
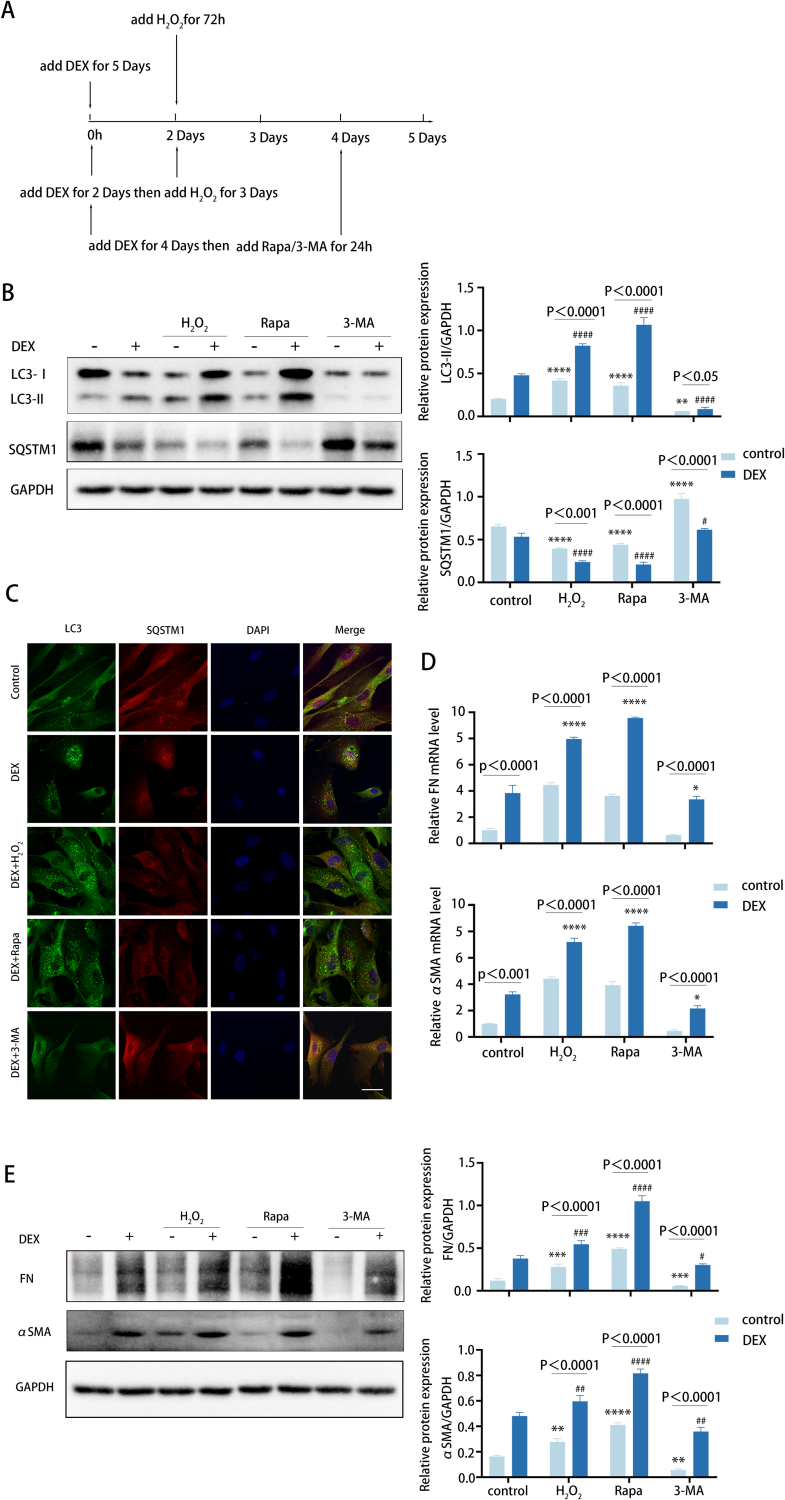
Autophagy inhibition attenuates fibrosis induced by DEX treatment. **(A)** Different treatments are described as follows. (i) Cells were treated with H_2_O_2_ (100 μM) for 72 h. (ii) Cells were treated with DEX (100 nM) for 5 days. (iii) Cells were treated with DEX for 2 days and then co-treated with H_2_O_2_ for 3 days. (iiii) Cells were treated with DEX (100 nM) for 4 days and then co-treated with Rapa (2 μM) or 3-MA (2 mM) for 24 h. **(B)** The protein levels of LC3 and SQSTM1 were detected by a Western blot. GAPDH was used as an internal control (left). Quantified protein levels were displayed as bar graphs (right). Data are presented as Mean ± SEM from three independent experiments (****p < 0.0001 vs. control group; #*p* < 0.05; ###*p* < 0.001 vs. DEX-treated group; one-way ANOVA). **(C)** Confocal images of LC3 (green), SQSTM1 (red), and DAPI (blue). Scale bar, 50 μm. **(D)** The mRNA expression levels of FN and αSMA were detected using RT-PCR. Data are presented as Mean ± SEM from three independent experiments (*P < 0.05, ****p < 0.0001 vs. DEX-treated group; one-way ANOVA). **(E)** The protein levels of FN and αSMA were detected by a Western blot. GAPDH was used as an internal control (left). Quantified protein levels were displayed as bar graphs (right). Data are presented as Mean ± SEM from three independent experiments (**p < 0.01, ***p < 0.001, ****p < 0.0001 vs. control group; #*p* < 0.05, ##*p* < 0.01, ###*p* < 0.001, ####*p* < 0.0001 vs. DEX-treated group; one-way ANOVA) Rapa, rapamycin; DEX, dexamethasone; 3-MA, 3-methyladenine.

To further verify the role of autophagy in TM fibrosis, we employed DEX to stimulate TM cells for 5 days to get an approximate model of POAG. For this purpose, TM cells were treated with DEX alone or in combination with H_2_O_2_, rapamycin, or 3-MA, respectively (Figure 5A).

We observed that DEX treatments increased the level of autophagy (Figures 5B,C). Meanwhile, pre-treatment with DEX led to higher autophagy levels following H_2_O_2_ treatment. Similarly, the presence of DEX enhanced the autophagic activity triggered by rapamycin (Figure 5B). As anticipated, DEX treatment dramatically elevated the expression levels of FN and αSMA, with further increases observed when DEX was combined with H_2_O_2_ or rapamycin at both mRNA and protein levels (Figures 5D,E). In contrast, treatment with 3-MA led to a decrease in FN and αSMA expression levels (Figure 5E). Therefore, these data suggest that DEX elevates autophagy to a certain level, which may contribute to fibrosis in the DEX-induced model. Of note, inhibited autophagy significantly attenuates fibrosis induced by DEX to some extent, indicating its potential role in clinical therapeutic intervention.

## 4 Discussion

In this study, we uncover the interplay between autophagy and the TGF-β2/Smad pathway in the pathogenesis of POAG. We observed increased autophagic activity in TM tissues from POAG patients and in TM tissues of aging healthy individuals. In human primary TM cells, we confirmed that autophagy becomes activated in the context of cellular senescence and the development of POAG, which further facilitates fibrotic progression via the TGF-β2/Smad signaling pathway. These findings underscore the important role of autophagy in POAG pathogenesis and confirm senescence as a pivotal risk factor.

To mimic the effects of autophagy on aging and POAG fibrosis *in vitro*, we used human primary TM cell cultures to establish an H_2_O_2_-induced aging model and a DEX-induced POAG model. Chronic oxidative stress effectively induces cellular senescence, making it a suitable model for studying the effects of aging on cells (37). We treated TM cells with 100 μM H_2_O_2_ and examined various senescent-associated biomarkers (Figures 2B–D). Our findings demonstrated an age-related increase in autophagy within the TM (Figure 2E), in align with the reported results on human TM tissues (13). Moreover, we treated TM cells with DEX for 5 days to establish an approximate model of POAG. This DEX-induced ocular hypertension (OHT) mimics POAG characteristics, including open angles and reduced outflow facility (41–43). As a well-established inducible model of outflow dysfunction, DEX-induced OHT could enhance our understanding of POAG (44). Both our aging and POAG models exhibited increased fibrosis, characterized by elevated levels of fibrotic markers FN and αSMA (Figures 5D,E). Importantly, our *in vitro* experiments confirmed the pivotal role of autophagy in TM fibrosis in both models (Figures 2E, 5E).

Autophagy has been demonstrated to play a major part throughout the entire POAG development, influencing both the modulation of IOP and the apoptosis of RGCs associated with elevated IOP. Autophagy exerts its impact on POAG by regulating IOP through its effects on TM cells. The Tg-MYOC^Y437H^ mouse model, known for its genetic predisposition to POAG, has shown that augmented autophagic flux effectively degrades the mutant myocilin protein within TM cells, thereby alleviating the increased IOP (45). Kristine Porter et al. also observed that TM cells isolated from POAG donors exhibit increased levels of phosphorylated mTOR, suggesting constitutive inhibition of autophagy (46). However, an important study recently provided direct evidence for the critical role of autophagy in glaucoma in an *in vivo* POAG model. Atg4b deficiency mitigates glaucomatous IOP elevation in the DBA/2 J mouse model, a well-established model for spontaneous glaucoma (47). In line with this study, our study revealed elevated autophagy activity in TM specimens derived from POAG patients compared to age-matched healthy controls (Figure 1A). Furthermore, our DEX-induced POAG model showed an increase in autophagy levels, concurrently with the augmentation of TM fibrosis (Figures 5B–E). Pathologically elevated IOP further triggers autophagy in RGCs, a response with dual roles in cell fate. Evidence is conflicting, with some studies suggesting a protective role for autophagy, while others imply it contributes to cell death. For instance, in a chronic glaucoma mouse model established by laser photocoagulation, upregulation of ATG3 to enhance autophagy was shown to induce RGC apoptosis (48). Additionally, the presence of a POAG-related mutation in the autophagy receptor OPTN has been shown to induce RGC death, and the knockdown of ATG5 has been observed to reduce RGC death, thus supporting the notion that autophagy can play a pro-death role in RGCs (49). Conversely, a study demonstrated that increased autophagy activity limited RGC death in an autophagy-positive mouse model, indicating a potential pro-survival function (50). In summary, autophagy is essential to the pathogenesis of POAG, as it affects the increase in IOP and the associated visual impairment.

As a homeostatic mechanism, autophagy is regulated by aging process, exhibiting variable activity levels across different ocular tissues and within the spectrum of age-related ocular disease. Our previous study indicated that lens epithelial cells (LECs) obtained from elder healthy donors demonstrated a more enduring activation of autophagy relative to those from younger healthy donors (51). The age-related decrease of autophagy within the retinal pigment epithelium (RPE) cells leads to lipofuscin accumulation, a consequence of incomplete photoreceptor outer segment digestion, which significantly contributes to the onset and progression of age-related macular degeneration (AMD) (52). Within the TM, the induction of autophagy in response to aging appears to be crucial for the maintenance of AH homeostasis (47). Consistent with this notion, our analysis of TM specimens from human biopsies, corroborated by the findings of Pulliero et al. (13), revealed that autophagy levels are elevated in older healthy individuals compared to their younger counterparts (Figure 1A). Using chronic H_2_O_2_-treated human TM cells, we observed that aging-induced autophagy activation, along with the accumulation of fibrotic proteins, may precipitate the development of POAG. This finding is supported by research conducted by Porter et al., which demonstrated that TM cells isolated from POAG patients exhibit elevated SA-*β*-Gal activity, a hallmark of cellular aging (46). Therefore, our findings confirm that aging, by triggering autophagy in TM cells, represents a significant risk factor in the development of POAG.

TGF-β2 is a pivotal cytokine that substantially promotes the fibrotic process that is hallmarked by an excessive accumulation of ECM components, culminating in the deterioration of normal organ function (53). A previous *in vivo* study has shown that lentiviral-mediated delivery of active TGF-*β*2 into the vitreous body leads to a progressive increase in IOP, which can be partially alleviated by the deficiency of autophagy (47). The findings demonstrate that IOP elevation induced by TGF-β2 may be regulated by autophagic processes, highlighting a potential relationship between these two pathways in the context of glaucoma pathogenesis. Our *in vitro* experiments have confirmed that autophagy promotes fibrosis in TM cells under conditions of aging and POAG, with this pro-fibrotic effect being primarily mediated by the TGF-β2/Smad signaling pathway (Figures 2E, 3E). Upon suppression of autophagy, we observed a decrease in the levels of pSmad3, FN, and αSMA levels, effects which were reversed by treatment with TGF-β2 (Figure 4B). Pretreatment of TM cells with a Smad phosphorylation inhibitor prevented the elevation of FN and αSMA protein levels induced by autophagy activation. These findings provide valuable insights into the mechanisms underlying autophagy-mediated fibrosis in TM cells. BAMBI, a negative regulator of TGF-β2 signaling that inhibits Smad3, is upregulated in autophagy-deficient TM cells and is primarily degraded via the autophagy-lysosomal pathway (54). Considering that BAMBI is downstream of TGF-β2 and our observations indicate that autophagy affects TGF-β2 at an upstream level, it is reasonable to deduce that autophagy might amplify TGF-β signaling through various mechanisms. We hypothesize that autophagy may promote fibrosis by degrading negative regulatory proteins that inhibit TGF-β2 synthesis or its receptor expression on the cell membrane, consequently boosting TGF-β2 secretion or amplifying cellular sensitivity to TGF-β2. In addition, TGF-β2 also enhanced the level of autophagy (Figures 4D,E). Collectively, these results further confirm the importance of autophagy in the pathology process of POAG, potentially creating a feed-forward loop that sustains and amplifies the fibrosis mediated by the TGF-β2/Smad pathway.

Overall, our study establishes a link between autophagy and the TGF-β2/Smad pathway in the pathogenesis of POAG. We found that aging, a significant factor, correlates with elevated autophagy that in turn induces TGF-β2 dependent fibrosis in the TM. Our work contributes to elucidating the role of autophagy in POAG development. Our work offers valuable knowledge for enhancing comprehension of fibrogenesis in POAG, uncovering the significance of autophagy. Intervention of autophagy may provide a potential strategy for the clinical POAG therapy.

## Data Availability

The original contributions presented in the study are included in the article/Supplementary material, further inquiries can be directed to the corresponding authors.
