# Supplementary material for: Aging-enhanced autophagy activity promotes fibrotic progression via the TGF-β2/Smad signaling pathway in trabecular meshwork cells—a new insight from POAG
Source: Front Med (Lausanne). 2025 Jan 15;11:1534120. doi: 10.3389/fmed.2024.1534120 (PMC11774994; doi:10.3389/fmed.2024.1534120)

Fig.2E

H_2O_2 100μM treated 1 time/day for 5 days

LC3B abcam(ab192890) 1:2000

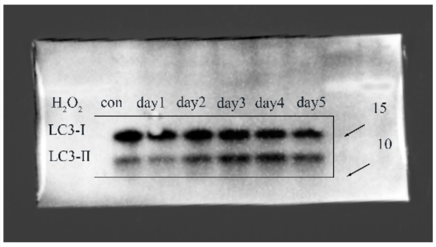

SQSTM1 abcam(ab67416) 1:2000

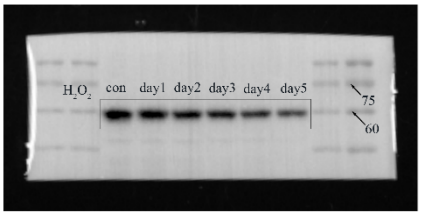

FN abcam(ab45688) 1:2000

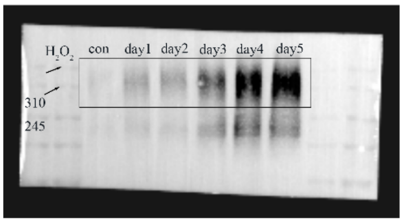

αSMA abcam(ab5694) 1:1000

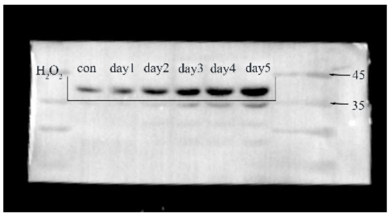

GAPDH ABclonal(A19056) 1:50000

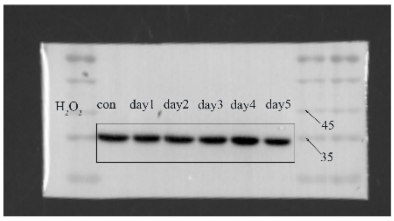

Fig.3B

LC3B abcam(ab192890) 1:2000

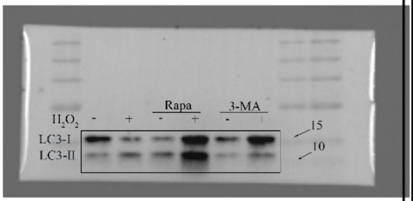

SQSTM1 abcam(ab67416) 1:2000

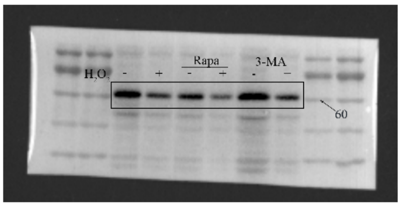

GAPDH ABclonal(A19056) 1:50000

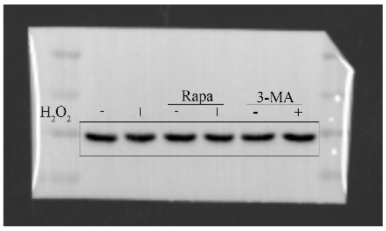

Fig.3E

FN abcam(ab45688) 1:2000

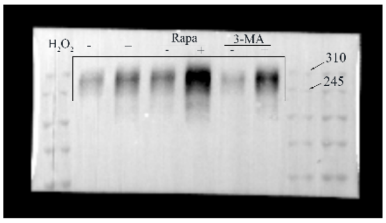

αSMA abcam(ab5694) 1:1000

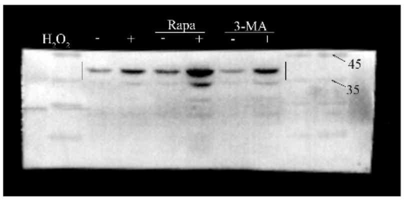

GAPDH ABclonal(A19056) 1:50000

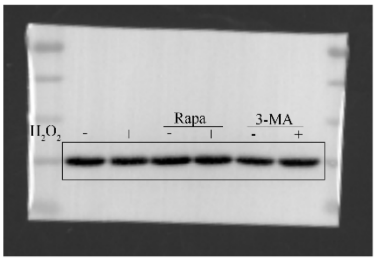

control: untreated

100μM H_2O_2 treated for 72h

100μM H_2O_2 treated for 48h then (2 μM)Rapa or (2 mM) 3-MA for 24h

treated with (2 μM)Rapa or (2 mM) 3-MA for 24h

Fig.4A

treated: treated with 0,0.5,1,2,5ng/ml TGF-β2

FN abcam(ab45688) 1:2000

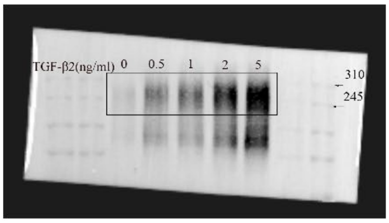

αSMA abcam(ab5694) 1:1000

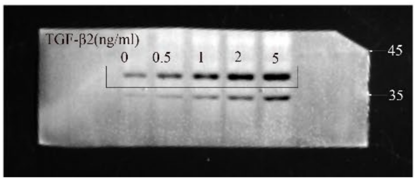

Smad3 abmart (T55013S) 1:1500

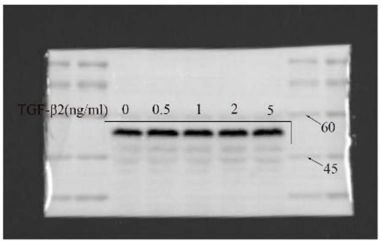

p-Smad3 abmart (T55140S) 1:1500

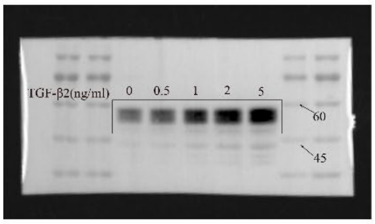

GAPDH ABclonal(A19056) 1:50000

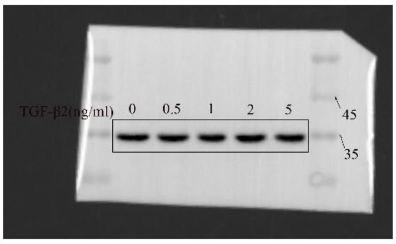

Fig.4B

treated with 3-MA for 24h

pre-treated with 3-MA for 24h then TGF-β2(5ng/ml) for 48h

Smad3 abmart (T55013S) 1:1500

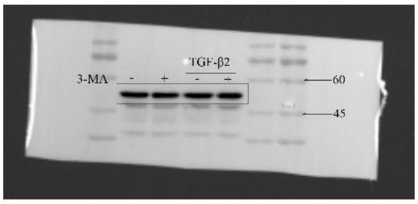

p-Smad3 abmart (T55140S) 1:1500

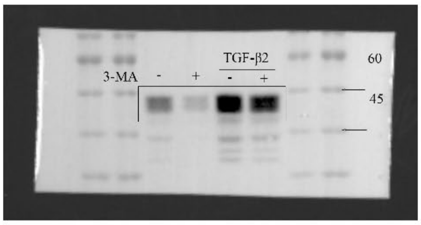

FN abcam(ab45688) 1:2000

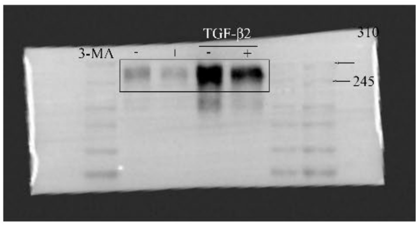

αSMA abcam(ab5694) 1:1000

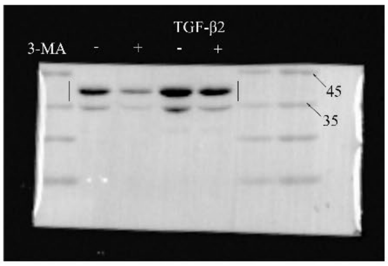

GAPDH ABclonal(A19056) 1:50000

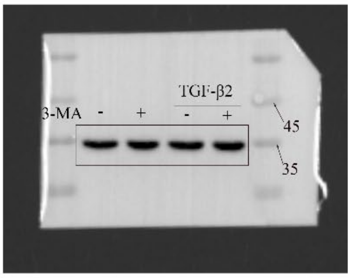

Fig.4C

pre-treated with SB-431542 for 2 h then 100μM H_2O_2 for 72h

pre-treated with SB-431542 for 2 h then added 100μM H_2O_2 for 48h then added Rapa for 24h

Smad3 abmart (T55013S) 1:1500

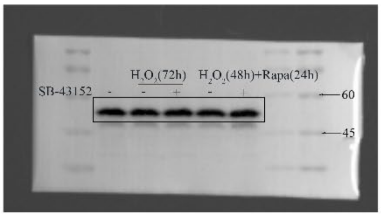

p-Smad3 abmart (T55140S) 1:1500

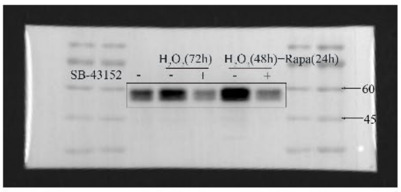

FN abcam(ab45688) 1:2000

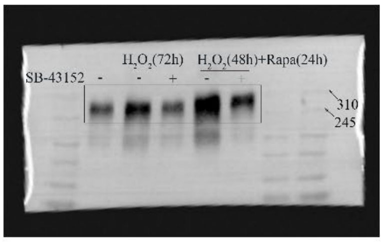

αSMA abcam(ab5694) 1:1000

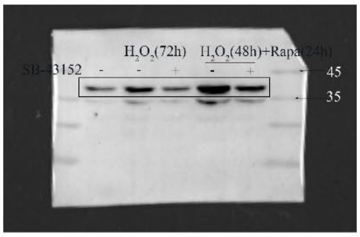

GAPDH ABclonal(A19056) 1:50000

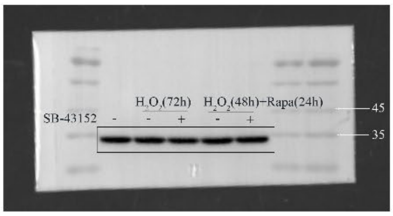

Fig.4D

control: untreated

treated: treated with TGF-β2(5ng/ml) for 48h

LC3B abcam(ab192890) 1:2000

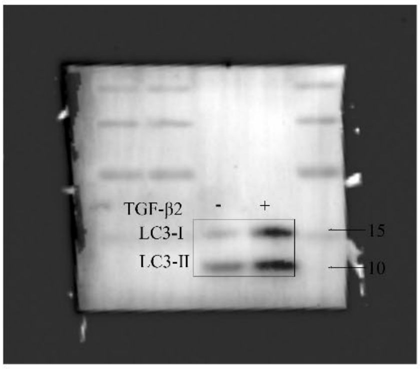

SQSTM1 abcam(ab67416) 1:2000

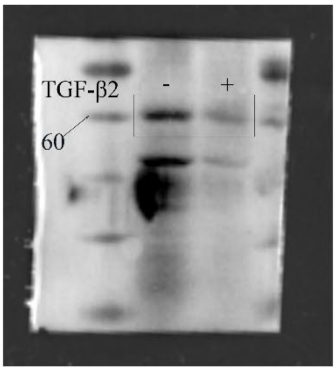

GAPDH ABclonal(A19056) 1:50000

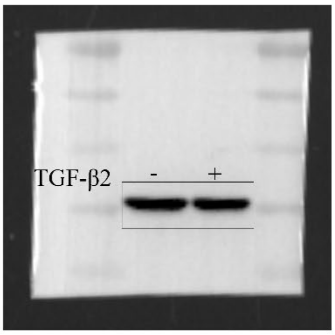

Fig.5B, 5E

normal control group: untreated

DEX-control group: 200μM DEX treated for 5 days

treated group 5: 200μM DEX treated for 2 days then 100μM H_2O_2 for 72h

treated group 6, 7: 200μM DEX treated for 4 days then (2 μM)Rapa or (2 mM)3-MA for 24h

LC3B abcam(ab192890) 1:2000

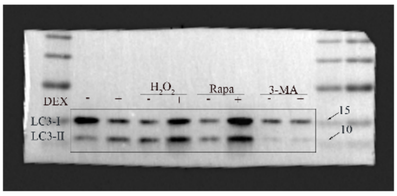

SQSTM1 abcam(ab67416) 1:2000

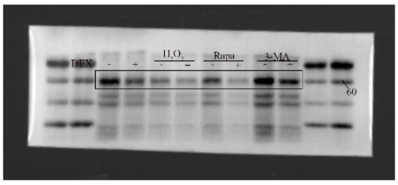

GAPDH ABclonal(A19056) 1:50000

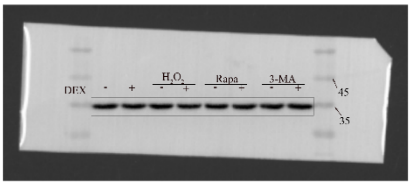

FN abcam(ab45688) 1:2000

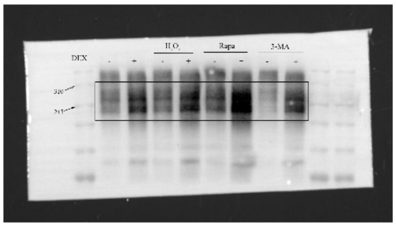

αSMA abcam(ab5694) 1:1000

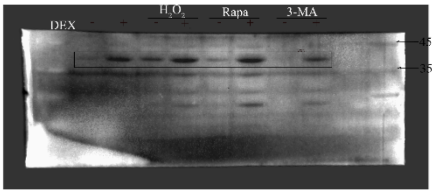

GAPDH ABclonal(A19056) 1:50000

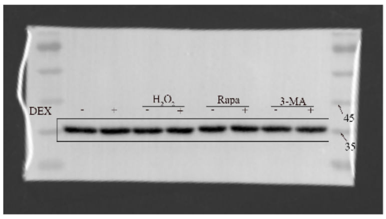

Supplement: Supplementary file 1 [file Data_Sheet_1.PDF]
